# Cerebral fuels within the first week of life in very preterm infants: a cohort study

**DOI:** 10.1136/archdischild-2025-328701

**Published:** 2025-10-13

**Authors:** Gordon Xin Hua Liu, Loredana Marcovecchio, K Beardsall, REACT Collaborative, Lynn Thomson

**Affiliations:** 1 School of Medicine, University of Auckland—Grafton Campus, Auckland, New Zealand; 2 Department of Paediatrics, University of Cambridge, Cambridge Biomedical Campus, Cambridge, UK; 3 Cambridge University Hospitals NHS Foundation Trust, Cambridge Biomedcal Campus, Cambridge, UK

**Keywords:** Neonatology, Endocrinology

## Abstract

**Background:**

Ketones and lactate may contribute towards overall cerebral fuel availability in term infants, yet the availability of such cerebral fuels in very preterm infants is unclear. We undertook a prespecified substudy to explore ketone and lactate concentrations in the first week of life in infants recruited to the REACT trial (real-time continuous glucose monitoring in the newborn): an international multicentre randomised controlled trial of 182 very low birth weight infants investigating the use of continuous glucose monitoring in glycaemic care.

**Methods:**

Ketone and lactate measurements were prospectively collected over the first week of life using the Nova Biomedical point-of-care meter. A longitudinal analysis was undertaken to explore lactate and ketone concentration trends across time and their relationships with blood glucose, baseline demographics, nutritional support and insulin treatment.

**Results:**

Data were available for 168 infants (85 females) including 2902 blood glucose, 2084 ketone and 2017 lactate samples. The mean (SD) gestational age was 27.4 (2.0) weeks. Lactate concentrations were higher initially, with mean (SD) 1.72 (1.26) mmol/L on day 2 and lowered to 1.19 (1.1) mmol/L on day 7. Ketone concentrations remained consistently low at 0.1 mmol/L. Neither simultaneous blood glucose concentrations, macronutrient intake nor receipt of insulin was consistently related to ketone or lactate concentrations.

**Conclusion:**

In this cohort of very preterm infants, there were persistently low concentrations of ketones and relatively higher concentrations of lactate throughout the first week of life. Future research should evaluate changes in these metabolites during episodes of acute hypoglycaemia or hyperglycaemia over more prolonged periods of neonatal intensive care.

**WHAT IS ALREADY KNOWN ON THIS TOPIC**

- During times of glycaemic instability, ketones and lactate may act as alternative cerebral fuels in healthy term infants, yet it is unclear if this occurs in preterm infants. We conducted a longitudinal analysis of very low birth weight infants over the first week of life exploring ketone and lactate trends and their association with blood glucose, baseline demographics, nutritional support and insulin treatment.

**WHAT THIS STUDY ADDS**

- We found that, in this cohort of very preterm infants, ketone concentrations remained persistently low while lactate concentrations were comparatively higher, which may reflect insufficient substrate availability and increased metabolic stress. There was no relationship between simultaneous blood glucose concentrations, increasing macronutrient support or receipt of insulin with ketone or lactate concentrations, although greater weight gain across the first week of life was associated with slightly greater lactic and ketogenic responses in later periods.

**HOW THIS STUDY MIGHT AFFECT RESEARCH, PRACTICE OR POLICY**

- This study provides insight into the presence of potential alternative cerebral fuels within a vulnerable population, and future studies should evaluate ketone and lactate profiles in preterm infants during periods of glucose instability.

## Introduction

 The neonatal period is characterised by significant glycaemic transition, driven by a cessation of exogenous glucose supply following cord cutting and the subsequent activation of glycogenolysis and gluconeogenesis to maintain blood glucose concentrations.^1 2^ These processes rely on mature enzymatic cascades and sufficient hepatic glycogen stores,^3 4^ and it can take up to 4 days in healthy term infants before adult glucose concentrations are reached. Ketogenesis and lactogenesis can supplement energy needs, particularly during times of increased energy demand or within hypoxic/glucose-deprived environments,^5^,^9^ and both ketones and lactate may contribute towards overall cerebral fuel availability in healthy term infants during the first 4 days of life.^7^

Prematurity (defined as <37 weeks gestational age) is a well-known risk factor for metabolic dysfunction.^3 4 10^ Preterm infants are at higher risk for both hypoglycaemia and hyperglycaemia compared with term counterparts secondary to hypoactive gluconeogenic pathways, inadequate glycogen reserves and abnormal insulin profiles.^3 4^ Moreover, physiological immaturity and instability, concomitant illness and painful procedures may further escalate energy demands, putting preterm infants into energy-deficient states that may adversely impact long-term outcomes.^5^

Although previous studies have attempted to characterise neonatal ketone and lactate profiles during the first few days of life,^78 11^,^15^ most are cross-sectional or have limited sample sizes and few have evaluated the impact of feeding or other nutritional parameters. Therefore, we aimed to explore glucose, ketone and lactate concentrations in a cohort of very preterm infants requiring intensive care in the first week of life. This was a prespecified substudy of infants recruited to the REACT trial (real-time continuous glucose monitoring in the newborn) with specific aims to explore trends over time, relationship to simultaneous glucose concentrations and impact of baseline demographics and nutritional support on plasma ketone and lactate concentrations.

## Methods

### Population

Data were collected as part of the REACT trial, which was an international, parallel-group, open-label randomised controlled trial (RCT). It was conducted in 13 neonatal intensive care units (NICUs) across the UK, Spain and the Netherlands between 2016 and 2019. Included infants had a birth weight of ≤1200 g, gestational age up to 33 weeks plus 6 days and were recruited within 24 hours of birth. Any infants with lethal congenital malformations or congenital metabolic disorders were excluded.

The protocol and findings for the REACT trial are available elsewhere.^16 17^ In brief, infants were randomised to receive glycaemic care that was informed by either real-time continuous interstitial glucose monitoring (CGM) (intervention) or standard care (control) in the first week of life. Infants in the intervention group were managed according to a tailored clinical management guideline based on the CGM data, which was read and recorded hourly, and advised that blood glucose concentrations should be measured at a minimum of every 12 hours or when there were rapid changes in CGM values or if CGM values fell to <4 mmol/L. Infants in the control group were managed according to local standard clinical practice with intermittent blood glucose concentrations guiding clinical management. All other aspects of clinical care, including nutritional intake, were at the discretion of local staff. All participants also had β-hydroxybutyrate (BOHB) and lactate concentrations measured using Nova Biomedical (Waltham, Massachusetts, USA) point-of-care (POC) devices at least once in a 24-hour period at the same time as blood glucose monitoring.

### Data collection

A case report form was completed at the end of each study day for the first week of life, which recorded details of clinical care including level of care, respiratory support, medications, nutritional intake and safety events. Weight was measured at birth and 7 days follow-up. SD scores (SDS) were calculated using the LLMS method^18^ according to the UK-WHO growth charts for preterm infants (British 1990 reference data, reanalysed 2009).^19^

### Assay methodology

Plasma glucose, BOHB and lactate measurements were performed using POC bedside metres (StatStrip Glucose/Ketone and StatStrip Lactate; Nova Biomedical). The StatStrip Glucose/Ketone metre requires 1.2 µL and 0.8 µL of blood for glucose and BOHB measurements, respectively, with measurement ranges of 0.6–33.3 mmol/L (glucose) and 0.0–7.0 mmol/L (BOHB).^20^ Coefficients of variability (CV) range from 4% (at 33.3 mmol/L) to 8% (at 2.8 mmol/L) for glucose, and 5% (at 7.0 mmol/L) to 7% (at 3.0 mmol/L) for BOHB. The StatStrip Lactate metre requires 0.6 µL of blood and has a measurement range of 0.3–20.0 mmol/L,^21^ with CV ranging from 1.7% (at 16.83 mmol/L) to 9.1% (at 0.76 mmol/L).

### Statistical analysis

Data are presented as n (%), mean (SD), and median (IQR) and were analysed in 24 hours epochs. Prior to performing our main analysis, we analysed BOHB and lactate concentrations between the intervention and control arms of the REACT trial to ensure that these values could be combined into a single cohort for analysis (online supplemental table 1). This analysis showed no significant difference across groups.

Glucose and lactate values were log(e)-transformed to near-normal distributions. BOHB values were predominantly left-skewed in our data set and so were categorised into ‘low’ (BOHB≤0.1 mmol/L) and ‘high’ (BOHB>0.1 mmol/L) subgroups during analysis. To account for repeated measures, analyses were conducted using generalised linear mixed regression models, which assumed normal distributions for glucose and lactate concentrations and binary logistic distributions for BOHB concentrations. Where appropriate, Holm-Bonferroni adjustment was used for multiple comparisons.

When assessing for differences in glucose, BOHB and lactate concentrations across epochs, day two was prespecified as the reference epoch. This was because we considered it to be the closest marker of baseline metabolite concentrations prior to significant clinical intervention, being the earliest time point when all participants had completed study enrolment and were consistently having metabolite concentrations measured.

We assessed the relationship between BOHB and lactate against glucose concentrations by comparing BOHB and lactate concentrations when simultaneous plasma glucose concentrations were in the lower, middle or upper tertile. Glucose tertile ranges were assigned based on the 33rd and 67th percentiles of all plasma glucose concentrations across the first week of life. We also compared BOHB and lactate concentrations across prespecified subgroups based on baseline demographics: sex (male vs female), gestation (<27 weeks vs ≥27 weeks), birth weight SDS (<−0.85 vs ≥−0.85), base excess at birth (<−6.16 vs ≥−6.16), intubation at birth (yes vs no) and receipt of maternal steroids (yes vs no).

To explore the impact of nutritional support, we calculated the cumulative total dose per kilogram of birth weight for carbohydrate, amino acid, lipid and breast milk supplementation and change in weight SDS score from birth to 7 days, and compared BOHB and lactate concentrations across cumulative doses of nutritional supplementation and weight change tertiles. The middle tertile was designated as the reference tertile. Preplanned analyses across formula supplementation tertiles were not possible due to the limited number of participants who received formula supplementation.

We analysed the effect of exogenous intravenous insulin dose by comparing BOHB and lactate concentrations against cumulative insulin dose tertiles, which were calculated by summating the total dose of insulin given across the first week of life and then categorising into lower, middle and high tertiles. We further assessed whether the effect of insulin varied according to age by conducting univariable analyses that compared BOHB and lactate concentrations across participants who did or did not receive insulin within a specific epoch. In epochs where a significant effect was demonstrated, multivariable analyses were then performed, with adjustment for the following pre-specified confounders: gestation, weight SDS from birth to 7 days, base excess at birth, intubation at birth, maternal steroids and simultaneous plasma glucose concentration.

We estimated total potential ATP availability from cerebral fuel metabolism by assigning an ATP equivalent to glucose (31), BOHB (21.5) and lactate (15).^7^

Analysis was conducted in R (V.4.3.1; R Core Team 2023). Percentile curves were constructed using the *purrr* and *ggplot* packages, and regression analysis using the *lmerTest* and *glmer* packages.

## Results

Of the 360 infants who were eligible for the REACT study, 180 (51%) were randomised, of which 168 (92%) had glucose, BOHB and/or lactate data recorded (n=167 glucose, 162 BOHB and 161 lactate). In total, we had 2902 glucose, 2084 BOHB and 2017 lactate samples, with the median (IQR) samples per infant being 16 (12–22) for glucose, 12 (8–16) for BOHB and 13 (8–16) for lactate.

For this cohort, the mean (SD) gestational age and birth weight were 27.4 (2.0) weeks and 890 (171) g, respectively. Half of our cohort were girls (n=85, 51%) and three-quarters were singletons (n=127, 76%). Delivery was by Caesarean section in 109 (65%) of infants and two-thirds of infants (n=105, 65%) were intubated at birth. The mean base excess at birth was −6.16. Further details on baseline demographics are provided in table 1.

**Table 1:** Maternal and neonatal baseline demographics

Demographic	n (N=168)	
Sex—female	168	85 (51)
Multiple birth	168	41 (24)
Ethnicity	168	
White		121 (72)
Black		13 (8)
Asian		17 (10)
Mixed		10 (6)
Other		7 (4)
Gestational age (weeks)	168	
Mean (SD)		27.4 (2.0)
Range		23.3–33.7
Received maternal steroids	168	129 (77)
Mode of delivery	168	
Spontaneous vaginal delivery		57 (34)
Caesarean section before onset of labour		74 (44)
Caesarean section after onset of labour		35 (21)
Assisted vaginal delivery		2 (1)
Birth weight (g)	168	890 (171)
Birth weight SD score	168	−0.85 (0.92)
Birth length (cm)	115	33.1 (2.5)
Head circumference (cm)	153	24.3 (1.8)
Apgar score		
1 min	160	5.39 (2.4)
5 min	161	7.64 (1.96)
Base excess at birth	165	−6.16 (5.2)
Intubation at birth	162	105 (65)

Values are n(%), mean (SD) or range.

### Time course of glucose, BOHB and lactate concentrations

**Figure 1:**
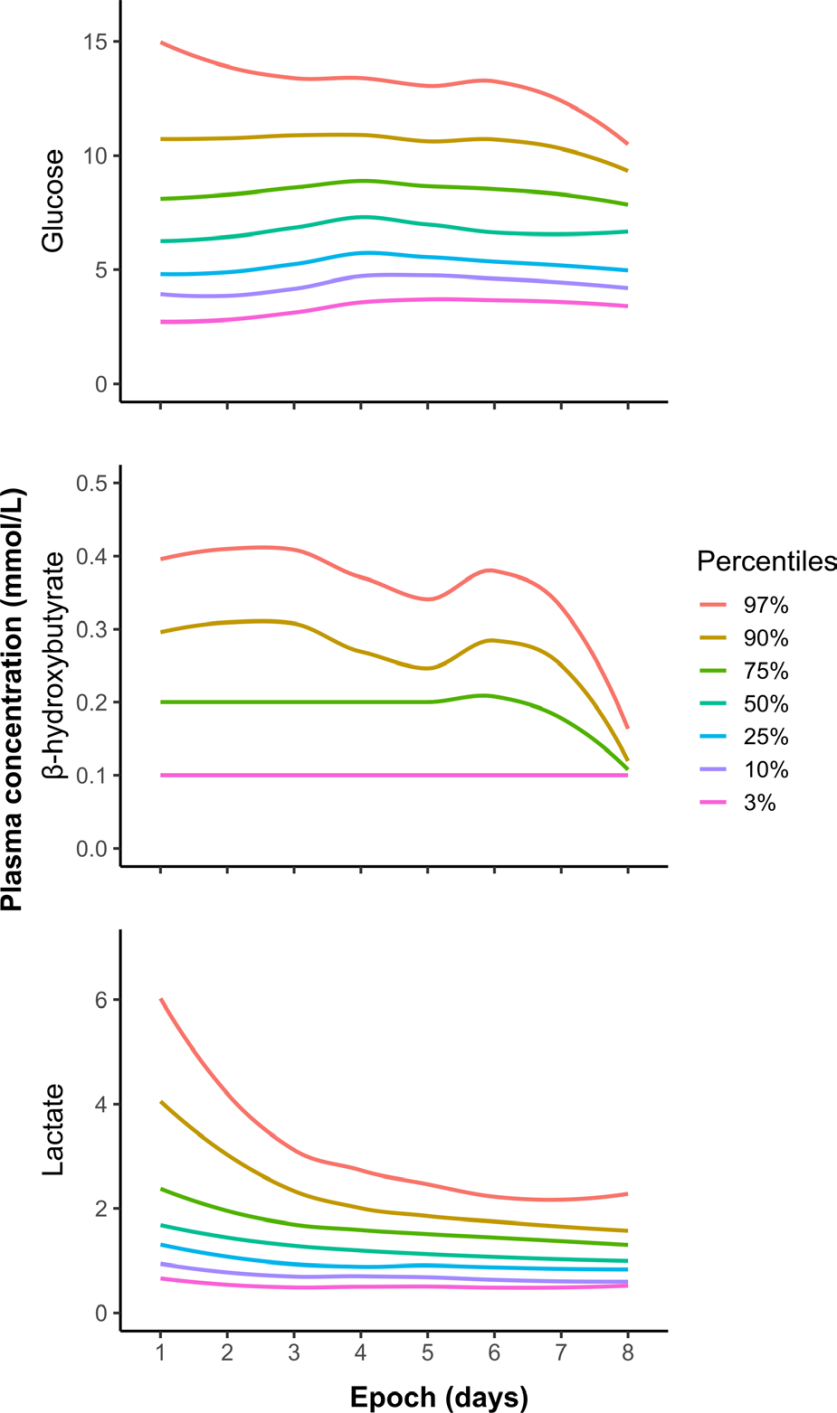
Percentile curves of plasma glucose, β-hydroxybutyrate and lactate concentrations across the first 8 days of life

The time course of glucose, BOHB and lactate concentration centiles is provided in figure 1. Plasma glucose concentrations were lower on day 2 (mean 6.5 mmol/L) but were consistently higher from days 3 to 7 (p<0.05), with mean concentrations ranging from 7.1 to 7.6 mmol/L (table 2). Only 24/2902 (1%) samples were in the hypoglycaemic range (<2.6 mmol/L). However, hyperglycaemia (defined as plasma glucose concentration >8.0 mmol/L) was common, with rates ranging from 21–37% per epoch. Lactate concentrations were higher on day 2 (mean 1.72 mmol/L) before falling steadily from days 3 to 7 (p<0.05), reaching mean values of 1.19 mmol/L on days 6 and 7. BOHB concentrations were low across all epochs, with the proportion of low BOHB readings (defined as BOHB≤0.1 mmol/L) increasing in later epochs (65% vs 75% on days 2 vs 5, respectively, p<0.05).

**Table 2:** Plasma concentrations of glucose, BOHB and lactate across different postnatal ages

Postnatal age (d)	Glucose (mmol/L)				BOHB (mmol/L)				Lactate (mmol/L)	
	Mean (SD)	n (%) of glucose readings <2.6 mmol/L	n (%) of glucose readings >8.0 mmol/L	N samples	Median (IQR)	BOHB ≤0.1 mmol/L	BOHB >0.1 mmol/L	N samples	Mean (SD)	N samples
1	7.1 (3.1)*	5 (3)	53 (29)	184	0.1 (0.1–0.2)	85 (66)	43 (34)	128	2.13 (1.46)*	126
2†	6.5 (3.0)	15 (3)	128 (23)	548	0.1 (0.1–0.2)	245 (65)	130 (35)	375	1.72 (1.26)	376
3	7.4 (2.8)*	5 (1)	167 (33)	501	0.1 (0.1–0.2)	243 (66)	124 (34)	367	1.51 (1.38)*	363
4	7.6 (2.6)*	2 (0.4)	170 (37)	461	0.1 (0.1–0.2)	233 (69)	104 (31)	338	1.33 (0.66)*	332
5	7.3 (2.4)*	0 (0)	148 (33)	457	0.1 (0.1–0.2)	246 (74)*	85 (26)	332	1.22 (0.51)*	332
6	7.2 (2.5)*	0 (0)	129 (31)	418	0.1 (0.1–0.2)	221 (72)	86 (28)	306	1.19 (0.50)*	302
7	7.1 (2.6)*	2 (1)	89 (30)	291	0.1 (0.1–0.2)	162 (74)	56 (26)	216	1.19 (1.1)*	217
8	6.5 (2.0)	0 (0)	9 (21)	42	0.1 (0.1–0.1)	21 (95)*	1 (5)	22	1.13 (0.51)*	23

Values are n (%), mean (SD) or median (IQR). P values are for comparisons of mean glucose and lactate values across postnatal age epochs using univariable linear mixed modelling or comparisons of low and high ketone values across postnatal age epochs using univariable binary logistic mixed modelling, reference epoch = ‘2’. Glucose and lactate values were log-transformed to near-normal distributions.

* p<0.05.

† Reference epoch.

BOHB, β-hydroxybutyrate.

Similar trends in glucose, BOHB and lactate concentrations across epochs were identified when we separated our cohort into intervention and control groups as per the original REACT trial (online supplemental table 2).

### BOHB and lactate concentrations in relation to glucose concentrations and baseline demographics

Lactate and BOHB concentrations were not consistently related to simultaneous glucose concentrations (table 3). Females and infants whose mothers had received steroids had higher proportions of low BOHB concentrations compared with males or infants who had not received maternal steroids (75% vs 65%, respectively, for sex, p=0.027; 72% vs 64%, respectively, for maternal steroids, p=0.038) (online supplemental table 3a). Meanwhile, mean lactate concentrations were higher in infants with lower versus higher birth weight SDS (1.59 vs 1.30 mmol/L, respectively, p=0.001) and a higher versus lower base excess at birth (1.59 vs 1.26 mmol/L, p<0.001) (online supplemental table 3b).

**Table 3:** Plasma concentrations of BOHB when simultaneous glucose concentrations were in lower, middle or upper tertile for that epoch

Age (d)	Glucose within tertile 1 (0.9–5.7 mmol/L)					Glucose within tertile 2 (5.7–7.9 mmol/L)*					Glucose within tertile 3 (7.9–24.1 mmol/L)				
	Ketones			Lactate (mmol/L)		Ketones			Lactate (mmol/L)		Ketones			Lactate (mmol/L)	
	BOHB ≤0.1 mmol/L	BOHB >0.1 mmol/L	N samples	Mean (SD)	N samples	BOHB ≤0.1 mmol/L	BOHB >0.1 mmol/L	N	Mean (SD)	N samples	BOHB ≤0.1 mmol/L	BOHB >0.1 mmol/L	N samples	Mean (SD)	N samples
1	33 (67)	16 (33)	49	2.12 (1.41)	48	32 (70)	14 (30)	46	2.15 (1.76)	42	20 (61)	13 (39)	33	2.03 (1.06)	32
2	117 (64)	66 (36)	183	1.36 (0.63)†	180	71 (70)	30 (30)	101	1.67 (0.96)	99	56 (64)	32 (36)	88	2.45 (2.00)	91
3	80 (73)	29 (27)	109	1.31 (0.76)	106	84 (67)	42 (33)	126	1.45 (1.08)	125	78 (61)	49 (39)	127	1.71 (1.96)	125
4	47 (73)	17 (27)	64	1.19 (0.57)	70	102 (70)	43 (30)	145	1.20 (0.45)	139	79 (65)	42 (35)	121	1.58 (0.85)†	116
5	68 (79)	18 (21)	86	1.16 (0.46)	92	97 (74)	34 (26)	131	1.22 (0.56)	129	70 (69)	32 (31)	102	1.29 (0.50)	97
6	81 (76)	26 (24)	107	1.09 (0.52)†	104	69 (66)	35 (34)	104	1.23 (0.42)	101	68 (74)	24 (26)	92	1.27 (0.54)	92
7	64 (80)	16 (20)	80	1.03 (0.52)	79	45 (74)	16 (26)	61	1.41 (1.82)	61	48 (69)	22 (31)	70	1.18 (0.71)	67
8	8 (89)	1 (11)	9	1.02 (0.48)	10	9 (100)	0 (0)	9	1.14 (0.64)	9	3 (100)	0 (0)	3	1.37 (0.21)	3

Values are n (%) or mean (SD). Lactate values were log-transformed to near-normal distribution during analysis. Glucose tertile ranges were assigned based on the 33rd and 67th percentiles of all plasma glucose concentrations across the first week of life. P values are for comparisons across groups within each specific epoch, with glucose tertile 2 being designated as the reference tertile and were obtained using separate univariable binary logistic mixed models for analysis of ketone concentrations and linear mixed models for analysis of lactate concentrations. P values are adjusted for multiple comparisons using Holm-Bonferroni correction.

* Reference tertile. n refers to the number of BOHB or lactate samples within that specific epoch and glucose tertile.

† Adjusted p<0.05 when compared against lactate value in glucose tertile 2.

BOHB, β-hydroxybutyrate.

### Impact of insulin and nutritional supplementation on BOHB and lactate concentrations

There was no consistent relationship between lactate and BOHB concentrations against receipt of insulin, both when analysed as cumulative dose tertiles (online supplemental table 4a and b) or when stratified across different epochs (table 4).

**Table 4:** Regression analyses assessing impact of insulin on plasma concentrations of BOHB and lactate within specific epochs

Postnatal age (d)	Insulin received				Insulin not received				P	
**BOHB**										
	**BOHB ≤0.1** **mmol/L**	**BOHB >0.1** **mmol/L**	**N babies**	**N samples**	**BOHB ≤0.1** **mmol/L**	**BOHB >0.1** **mmol/L**	**N babies**	**N samples**	**Univariable**	Multivariable*†
1	22 (71)	9 (29)	17	31	63 (65)	34 (35)	61	97	0.664	N/A
2	67 (61)	43 (39)	37	110	172 (66)	87 (34)	108	259	0.664	N/A
3	87 (61)	56 (39)	48	143	153 (69)	68 (31)	99	221	0.497	N/A
4	97 (65)	52 (35)	51	149	132 (72)	52 (28)	90	184	0.664	N/A
5	122 (71)	50 (29)	55	172	121 (78)	34 (22)	74	155	0.664	N/A
6	100 (64)	56 (36)	51	156	118 (80)	30 (20)	73	148	0.198	N/A
7	67 (64)	38 (36)	38	105	93 (84)	18 (16)	65	111	0.027	0.001
8	9 (90)	1 (10)	10	10	12 (100)	0 (0)	11	12	0.052	N/A
**Lactate**										
	**Mean (SD**)		**N babies**	**N samples**	**Mean (SD**)		**N babies**	**N samples**	**Univariable**	Multivariable*†
1	1.99 (0.860)		18	31	2.18 (1.60)		57	95	1.00	N/A
2	2.19 (1.85)		38	113	1.52 (0.821)		106	257	0.020	0.403
3	1.75 (2.01)		49	144	1.35 (0.696)		97	216	0.253	N/A
4	1.51 (0.798)		48	139	1.21 (0.509)		89	188	0.005	0.209
5	1.29 (0.556)		55	168	1.15 (0.449)		76	159	0.750	N/A
6	1.23 (0.504)		51	153	1.15 (0.493)		73	147	0.750	N/A
7	1.19 (1.33)		41	104	1.20 (0.841)		66	112	1.00	N/A
8	1.29 (0.593)		9	9	1.02 (0.439)		13	14	0.750	N/A

Values are n (%) or mean (SD). P values are for comparisons across groups within each specific epoch and were obtained using separate binary logistic mixed models for analysis of BOHB concentrations and separate linear mixed models for analysis of lactate concentrations.

P values for univariable analyses are adjusted for multiple comparisons using Holm-Bonferroni correction.

* Epochs that indicated a significant effect of insulin on plasma BOHB or lactate concentrations were subsequently selected for multivariable analysis.

† Multivariable analyses were adjusted for the following confounders: gestation, weight SD score from birth to 7 days, base excess at birth, intubation at birth, maternal steroids and simultaneous plasma glucose concentration.

BOHB, β-hydroxybutyrate; N/A, not assessed.

When compared against the middle tertile, higher cumulative volumes of breast milk were associated with a higher proportion of low BOHB concentrations (70% vs 75%, respectively) and higher lactate concentrations (1.28 vs 1.59 mmol/L, respectively, p<0.05) (online supplemental table 4a and b). Conversely, greater increases in weight SDS were associated with a lower proportion of low BOHB concentrations when compared with the middle tertile (67% vs 74%, respectively, p<0.05) and higher lactate concentrations (1.57 vs 1.34 mmol/L, p<0.05). There was no relationship between BOHB and lactate concentrations with carbohydrate, amino acid or lipid supplementation dose.

### ATP contribution or glucose, BOHB and lactate

Glucose was the predominant ATP source across all epochs, contributing between 90% and 95% (online supplemental table 5). Lactate initially provided 8–9% of overall ATP during days 1–2, although this halved to 4–5% by days 7–8. BOHB comprised approximately 1% of total ATP across epochs.

## Discussion

This study is the first to characterise the concentrations of BOHB and lactate in very preterm infants across the first week of life. Our findings indicate that very preterm infants do not have significant circulating concentrations of BOHB or lactate during times of significant physiological stress and must therefore rely predominantly on glucose as their primary energy source. Increasing macronutrient support or receipt of insulin was not associated with different BOHB or lactate concentrations, although greater weight gain may influence BOHB and lactate concentrations in later stages. We also found that female sex, lower birth weight, lower base excess at birth and receipt of maternal steroids were associated with higher concentrations of BOHB or lactate.

The neonatal brain derives approximately 70% of its energy needs from glucose.^22^ Ketones and lactates are produced via β-oxidation of free fatty acids or anaerobic fermentation of glucose respectively^23 24^ and, alongside other roles as signalling molecules,^24^,^27^ can be used to supplement energy needs during times of increased demand or insufficient supply by acting as oxidative substrates. As such, ketones and lactate may play important roles during times of stress or disturbed glucose metabolism,^27^,^29^ such as following traumatic brain injury or periods of fasting.^30 31^ Previous research has suggested that extremely preterm infants are prone to states of relative insulin deficiency,^32^ secondary to both reduced secretion of and sensitivity to insulin, particularly in the immediate postnatal phase when there is increased catabolic stress.^33^ These changes are partially demonstrated in our cohort, where the prevalence of hypoglycaemia and hyperglycaemia was low and high respectively, likely reflecting the care received in NICU. Our finding that receipt of insulin was not associated with significant differences in lactate or BOHB concentrations further reflects the underlying insulin-resistant state that persisted in our cohort across the immediate postnatal period.

The metabolic frailty of our cohort is further exemplified when BOHB and lactate concentrations are compared against previously reported data from term counterparts. Overall, our cohort had consistently low concentrations of BOHB with relatively higher concentrations of lactate. The Glucose in well babies (GLOW) study, which analysed alternative fuel concentrations in healthy term babies across the first 5 days of life, reported similar median concentrations of lactate (1.9 mmol/L in day 1 to 1.4 mmol/L in day 5) but significantly higher BOHB concentrations (ranging from 0.1 to 0.7 mmol/L).^7^ Given our cohort’s extreme prematurity and low birth weight, it is probable that our cohort lacked sufficient energy stores to mount appropriate ketogenic responses (and therefore had low BOHB concentrations), whereas the relatively higher lactate concentrations reflected greater degrees of metabolic stress and anaerobic respiration. This is supported by our finding that lower birth weight and a more negative base excess at birth, reflective of poorer metabolic condition, were both associated with higher lactate concentrations and is consistent with previous research that has suggested preterm babies as being at higher risk for hypoglycaemic injury secondary to insufficient fat and protein stores.^3 6^

Interestingly, we found that greater weight gain from birth to 7 days was associated with higher lactate and ketone concentrations, which may reflect how our infants had had more time by then to generate sufficient energy reserves and increase their sensitivity towards exogenous insulin. However, we did not find any association between greater macronutrient support and higher concentrations of BOHB or lactate, suggesting that this slight increase in ketogenic and lactic response may not be attributable to a specific nutrient source, but is related to a general accretion of tissue and body mass. Alternatively, our observed low BOHB concentrations and lack of effect of nutritional supplementation may also be related to the relatively immaturity of enzymatic pathways in preterm infants compared with term counterparts, which may limit the rate or capacity for ketogenesis and lactogenesis to be performed.

To our knowledge, only two previous studies have assessed alternative fuel concentrations in preterm infants.^8 13^ Hawdon *et al*^13^ found that, during the first postnatal week, very preterm infants had low concentrations of ketone bodies compared with term counterparts (mean 0.04 vs 0.20 mmol/L, respectively), and that lower gestational age (particularly before 36 weeks) was a significant predictor of reduced ketone body concentrations, even after adjustment for birth size, feeding variables and blood glucose concentrations. In contrast to our findings, they also reported that increased volume of enteral feeding was positively correlated with ketone body concentrations. However, their cross-sectional analysis only included clinically stable preterm participants, who likely had more robust energy reserves and were subjected to less metabolic stress than our cohort of unwell infants.

This study employed recent developments in POC technology to allow bedside collection of small samples in a vulnerable population. A recent systematic review assessing the utility of POC BOHB measurements in emergency departments for people without diabetes found that most of the available data relates to children with gastroenteritis, and that there is limited information that is specific for other paediatric populations.^34^ Nonetheless, these metres are widely used clinically,^34^ and POC BOHB measurements have been previously validated in unwell preterm and term infants.^35^ Further, their ease of use and low volume of blood required allows for repeated samples in infants with limited circulating volumes and enables clinicians to serially monitor metabolites that would otherwise only be intermittently measured.

Limitations of this study include the limited data at low blood glucose concentrations, which may contribute towards lower BOHB and lactate concentrations, and that we had relatively fewer samples during the first 24 hours of life, which has been recognised as a time of significant metabolic transition.^2^ It is also important to recognise that our data was collected as part of a larger RCT assessing glycaemic control, which may alter clinician behaviour and restrict generalisability of our findings towards standard clinical care. Strengths of our study include its multicentric nature, longitudinal design spanning several days, large sample size and detailed collection of clinical care and nutritional data.

In conclusion, we report that very and extremely preterm infants in NICU have low concentrations of BOHB with relatively higher lactate concentrations, which may reflect insufficient substrate availability and increased metabolic stress. We did not find any effect of increased macronutrient support, breastfeeding or receipt of insulin on lactate or BOHB concentrations, although weight gain appeared to increase ketogenic and lactic responses in later periods. Future studies should continue to explore the metabolic profiles of this vulnerable population during periods of glycaemic instability (eg, hypoglycaemias or hyperglycaemias) and whether their trajectories can be altered using clinical interventions.

## Supplementary material

10.1136/archdischild-2025-328701 online supplemental file 1

## Data Availability

Data are available upon reasonable request.
