# Supplementary material for: Cerebral fuels within the first week of life in very preterm infants: a cohort study
Source: Arch Dis Child Fetal Neonatal Ed. 2025 Oct 13;111(3):e328701. doi: 10.1136/archdischild-2025-328701 (PMC13151478; doi:10.1136/archdischild-2025-328701)
Supplement: online supplemental file 1 [file fetalneonatal-111-3-s001.pdf]

# Supplementary files

## Supplementary table 1: Plasma concentrations of BOHB and lactate across different postnatal ages between intervention and control arms of the REACT trial

| Postnatal age (d) | BOHB (mmol/L) | | | | p* |
|---|---|---|---|---|---|
| | Intervention | n samples | Control | n samples | |
| **BOHB** | | | | | |
| 1 | 0.1 (0.1, 0.2) | 69 | 0.1 (0.1, 0.2) | 59 | 1.00 |
| 2 | 0.1 (0.1, 0.2) | 184 | 0.1 (0.1, 0.2) | 191 | 1.00 |
| 3 | 0.1 (0.1, 0.2) | 184 | 0.1 (0.1, 0.2) | 183 | 1.00 |
| 4 | 0.1 (0.1, 0.2) | 171 | 0.1 (0.1, 0.2) | 166 | 1.00 |
| 5 | 0.1 (0.1, 0.1) | 168 | 0.1 (0.1, 0.2) | 163 | 0.722 |
| 6 | 0.1 (0.1, 0.2) | 151 | 0.1 (0.1, 0.1) | 156 | 1.00 |
| 7 | 0.1 (0.1, 0.2) | 107 | 0.1 (0.1, 0.1) | 111 | 1.00 |
| 8 | 0.1 (0.1, 0.1) | 8 | 0.1 (0.1, 0.1) | 14 | 1.00 |
| **Lactate†** | | | | | |
| 1 | 2.10 (1.50) | 64 | 2.16 (1.42) | 2.16 | 1.00 |
| 2 | 1.62 (1.19) | 190 | 1.83 (1.32) | 186 | 0.948 |
| 3 | 1.40 (0.91) | 185 | 1.62 (1.74) | 178 | 0.948 |
| 4 | 1.30 (0.745) | 163 | 1.36 (0.570) | 168 | 0.868 |
| 5 | 1.19 (0.527) | 169 | 1.25 (0.491) | 162 | 0.732 |
| 6 | 1.14 (0.415) | 145 | 1.24 (0.559) | 158 | 0.948 |
| 7 | 1.23 (1.43) | 105 | 1.15 (0.657) | 113 | 1.00 |
| 8 | 1.18 (0.454) | 10 | 1.08 (0.563) | 13 | 1.00 |

*Values are median (IQR). P-values are for comparisons across intervention and control arms using Mann-Whitney-U test unless indicated otherwise. †Lactate values were log-transformed to near-normal distributions and compared using independent 2-sided t-tests. *p-values were adjusted for multiple comparisons using Holm-Bonferroni correction. BOHB: β-hydroxybutyrate. REACT: Real Time Continuous Glucose Monitoring in Neonatal Intensive Care trial.*

**Supplementary table 2: Plasma concentrations of glucose, BOHB, and lactate across different postnatal ages between intervention and control arms of the REACT trial**

| Postnatal age (d) | Glucose (mmol/L) | | BOHB (mmol/L) | | | Lactate (mmol/L) | |
|---|---|---|---|---|---|---|---|
| | Mean (SD) | N samples | BOHB ≤0.1mmol/L | BOHB >0.1mmol/L | N samples | Mean (SD) | N samples |
| **Intervention** | | | | | | | |
| 1 | 7.4 (3.2)* | 101 | 43 (62%) | 26 (38%) | 69 | 2.1 (1.5)* | 64 |
| 2† | 6.2 (2.8) | 291 | 122 (64%) | 69 (36%) | 191 | 1.6 (1.2) | 190 |
| 3 | 7.03 (2.3)* | 263 | 125 (68%) | 59 (32%) | 184 | 1.4 (0.9)* | 185 |
| 4 | 7.4 (2.3)* | 232 | 118 (69%) | 53 (31%) | 171 | 1.3 (0.8)* | 163 |
| 5 | 6.7 (1.9)* | 233 | 131 (78%)* | 37 (22%) | 168 | 1.2 (0.5)* | 169 |
| 6 | 6.5 (1.8)* | 206 | 103 (68%) | 48 (32%) | 151 | 1.1 (0.4)* | 145 |
| 7 | 6.3 (1.9)* | 148 | 77 (72%) | 30 (28%) | 107 | 1.2 (1.4)* | 105 |
| 8 | 5.5 (1.8) | 18 | 7 (88%) | 1 (13%) | 8 | 1.2 (0.5) | 10 |
| **Control** | | | | | | | |
| 1 | 6.7 (3.0) | 83 | 42 (71%) | 17 (29%) | 59 | 2.2 (1.4) | 62* |
| 2† | 6.8 (3.2) | 257 | 123 (67%) | 61 (33%) | 184 | 1.8 (1.3) | 186 |

| | | | | | | | |
|---|---|---|---|---|---|---|---|
| 3 | 7.7 (3.2)* | 238 | 118 (65%) | 65 (36%) | 183 | 1.6 (1.7) | 178* |
| 4 | 7.8 (2.9)* | 228 | 115 (69%) | 51 (31%) | 166 | 1.4 (0.6) | 168* |
| 5 | 7.9 (2.8)* | 223 | 115 (71%) | 48 (29%) | 163 | 1.3 (0.5) | 162* |
| 6 | 7.9 (2.8)* | 213 | 118 (76%) | 38 (24%) | 156 | 1.2 (0.6) | 158* |
| 7 | 7.9 (3.0)* | 145 | 85 (77%) | 26 (23%) | 111 | 1.2 (0.7) | 113* |
| 8 | 7.3 (1.8) | 24 | 14 (100%) | 0 (0%) | 14 | 1.1 (0.6) | 13* |

*Values are n (%), mean (SD), or median (IQR). P-values are for comparisons of mean glucose and lactate values across postnatal age epochs using univariable linear mixed modelling or comparisons of low and high ketone values across postnatal age epochs using univariable binary logistic mixed modelling, reference epoch = "2". Glucose and lactate values were log-transformed to near-normal distributions. *p<0.05. †Reference epoch. BOHB: β-hydroxybutyrate. IQR: Interquartile range. SD: Standard deviation*

## Supplementary table 3a: Plasma concentrations of BOHB across infants with different baseline demographics

| Variable | Yes | | | No | | | p |
|---|---|---|---|---|---|---|---|
| | BOHB ≤0.1 mmol/L | BOHB >0.1 mmol/L | N samples | BOHB ≤0.1 mmol/L | BOHB >0.1 mmol/L | N samples | |
| Female | 757 (75%) | 259 (26%) | 1016 | 699 (65%) | 370 (35%) | 1069 | 0.027 |
| Gestation < 27 weeks | 622 (65%) | 331 (35%) | 953 | 834 (74%) | 298 (26%) | 1132 | 0.071 |
| Birthweight SDS < -0.85 | 636 (71%) | 259 (29%) | 895 | 820 (69%) | 270 (31%) | 1190 | 0.575 |
| Base excess at birth < -6.16 | 697 (68%) | 322 (32%) | 1019 | 739 (71%) | 297 (29%) | 1036 | 0.231 |
| Intubation at birth | 812 (64%) | 449 (36%) | 1261 | 545 (76%) | 176 (24%) | 721 | 0.097 |
| Received maternal steroids | 1115 (72%) | 433 (28%) | 1548 | 341 (64%) | 196 (37%) | 537 | 0.038 |

*Values are n (%). p-values are for comparisons across groups and obtained using univariable binary logistic mixed modelling. BOHB: β-hydroxybutyrate. SDS: Standard deviation score.*

## Supplementary table 3b: Plasma concentrations of lactate across infants with different baseline demographics

| Variable | Yes | | No | | p |
|---|---|---|---|---|---|
| | Lactate (mmol/L) | n samples | Lactate (mmol/L) | n samples | |
| **Lactate** | | | | | |
| Female | 1.40 (1.02) | 1027 | 1.45 (1.07) | 1044 | 0.671 |
| Gestation < 27 weeks | 1.37 (1.08) | 937 | 1.47 (1.02) | 1134 | 0.330 |
| Birthweight SDS < -0.85 | 1.59 (1.14) | 919 | 1.30 (0.949) | 1152 | 0.001 |
| Base excess at birth < -6.16 | 1.59 (1.25) | 1042 | 1.26 (0.759) | 999 | <0.001 |
| Intubation at birth | 1.45 (1.10) | 1265 | 1.40 (0.989) | 704 | 0.667 |
| Received maternal steroids | 1.42 (1.07) | 1527 | 1.44 (0.993) | 544 | 0.839 |

*Values are mean (SD). Lactate values were log-transformed to near-normal distribution during analysis. p-values are for comparisons across groups and obtained using univariable linear mixed modelling. SDS: Standard deviation score.*

## Supplementary table 4a: Cumulative impact of nutritional support, insulin, and total weight change on plasma concentrations of BOHB

| Intervention | Intervention amount within tertile 1 | | | | Intervention amount within tertile 2[‖] | | | | Intervention amount within tertile 3 | | | |
|---|---|---|---|---|---|---|---|---|---|---|---|---|
| | Cumulative dose range (g/kg)† | BOHB ≤0.1 mmol/L | BOHB >0.1 mmol/L | N samples | Cumulative dose range (g/kg)† | BOHB ≤0.1 mmol/L | BOHB >0.1 mmol/L | N samples | Cumulative dose range (g/kg)† | BOHB ≤0.1 mmol/L | BOHB >0.1 mmol/L | N samples |
| Carbohydrates | 4.29 to 58.7 | 357 (71%) | 146 (29%) | 503 | 58.7 to 74.4 | 542 (67%) | 272 (33%) | 814 | 74.4 to 110 | 557 (73%) | 211 (28%) | 768 |
| Amino acids | 2.59 to 1470 | 350 (64%) | 194 (36%) | 544 | 1470 to 2000 | 563 (74%) | 203 (27%) | 766 | 2000 to 4320 | 482 (68%) | 224 (32%) | 706 |
| Lipids | 0.05 to 10.0 | 413 (70%) | 177 (30%) | 590 | 10.0 to 14.2 | 541 (67%) | 258 (32%) | 799 | 14.2 to 26.2 | 487 (72%) | 193 (28%) | 680 |
| Breast milk‡ | 1.65 to 35.6 | 384 (68%) | 178 (32%) | 562 | 35.6 to 131 | 528 (70%) | 231 (30%) | 759 | 131 to 661* | 428 (75%) | 140 (25%) | 568 |
| Insulin[§] | 0.00 to 3.37 | 200 (70%) | 86 (30%) | 286 | 3.37 to 6.98 | 321 (65%) | 177 (36%) | 498 | 6.98 to 139 | 277 (62%) | 173 (38%) | 450 |
| Weight SDS change[¶] | -1.49 to -0.589 | 450 (69%) | 204 (31%) | 654 | -0.589 to -0.299 | 527 (74%) | 185 (26%) | 712 | -0.299 to 0.612* | 452 (67%) | 224 (33%) | 676 |

*Values are n (%). Tertiles categorised according to cumulative dose ranges: 1 (0% – 33% percentile), 2 (33% – 67% percentile), 3 (67% - 100% percentile). p-values are for comparisons across groups, with tertile 2 being designated as the reference tertile, and obtained using univariable mixed linear modelling. *p<0.05 when compared against tertile 2. ‖Reference tertile. †This reflects cumulative dose range and units are g/kg of birthweight unless stated otherwise. ‡This reflects cumulative volume range and units are mls/kg of birthweight. §Units are IU/kg of birthweight. ¶This reflects weight SDS change from birthweight to weight at 7-days. BOHB: β-hydroxybutyrate. IU: International units. SDS: Standard deviation score.*

## Supplementary table 4b: Cumulative impact of nutritional support, insulin, and total weight change on plasma concentrations of lactate

| Intervention | Intervention amount within tertile 1 | | | Intervention amount within tertile 2[‖] | | | Intervention amount within tertile 3 | | |
|---|---|---|---|---|---|---|---|---|---|
| | Cumulative dose range (g/kg)† | Lactate (mmol/L) | n samples | Cumulative dose range (g/kg)† | Lactate (mmol/L) | n samples | Cumulative dose range (g/kg)† | Lactate (mmol/L) | n samples |
| Carbohydrates | 4.29 to 58.7 | 1.38 (0.997) | 489 | 58.7 to 74.4 | 1.43 (1.18) | 802 | 74.4 to 110 | 1.46 (0.928) | 780 |
| Amino acids | 2.59 to 1470 | 1.46 (1.06) | 546 | 1470 to 2000 | 1.40 (1.06) | 751 | 2000 to 4320 | 1.44 (1.06) | 713 |
| Lipids | 0.05 to 10.0 | 1.50 (1.17) | 601 | 10.0 to 14.2 | 1.46 (1.06) | 772 | 14.2 to 26.2 | 1.32 (0.906) | 682 |
| Breast milk‡ | 1.65 to 35.6 | 1.59 (1.27)* | 586 | 35.6 to 131 | 1.28 (0.864) | 747 | 131 to 661 | 1.22 (0.561) | 538 |
| Insulin[§] | 0.00 to 3.37 | 1.53 (1.20) | 307 | 3.37 to 6.98 | 1.43 (1.07) | 470 | 6.98 to 139 | 1.61 (1.34) | 461 |
| Weight SDS change[¶] | -1.49 to -0.589 | 1.34 (0.721) | 635 | -0.589 to -0.299 | 1.34 (1.15) | 697 | -0.299 to 0.612 | 1.57 (1.03)* | 700 |

*Values are mean (SD). Lactate values were log-transformed to near-normal distribution during analysis. Tertiles categorised according to cumulative dose ranges: 1 (0% – 33% percentile), 2 (33% – 67% percentile), 3 (67% - 100% percentile). p-values are for comparisons across groups, with tertile 2 being designated as the reference tertile, and obtained using univariable linear mixed modelling. *p<0.05 when compared against tertile 2. [‖]Reference tertile. †This reflects cumulative dose range and units are g/kg of birthweight unless stated otherwise. ‡This reflects cumulative volume range and units are mls/kg of*

*birthweight. §Units are IU/kg of birthweight. ¶This reflects weight SDS change from birthweight to weight at 7-days. IU: International units. SDS: Standard deviation score.*

## Supplementary table 5: ATP equivalents for glucose, BOHB, and lactate across different postnatal ages

| Postnatal age (d) | N | | Percent of total ATP | | | Total ATP (mmol/L) |
|---|---|---|---|---|---|---|
| | Samples | Infants | Glucose | BOHB | Lactate | |
| 1 | 188 | 94 | 90.1 | 1.0 | 9.0 | 238 (7.4) |
| 2 | 555 | 160 | 90.8 | 1.2 | 8.0 | 218 (4.3) |
| 3 | 510 | 156 | 92.3 | 1.1 | 6.6 | 242 (4.2) |
| 4 | 471 | 153 | 93.2 | 1.1 | 5.7 | 247 (4.1) |
| 5 | 471 | 143 | 93.6 | 0.9 | 5.5 | 234 (3.9) |
| 6 | 426 | 143 | 93.5 | 1.1 | 5.4 | 235 (4.0) |
| 7 | 307 | 133 | 93.3 | 1.1 | 5.7 | 225 (5.2) |
| 8 | 43 | 31 | 95.1 | 0.5 | 4.4 | 208 (10.3) |

*Values are number or mean (SE). ATP equivalents assumed to be 31 mmol ATP per 180g (1mmol) of glucose, 21.5mmol ATP per 104g (1 mmol) of BOHB, and 15mmol ATP per 89g (1mmol) of lactate.(7) ATP: Adenosine triphosphate. BOHB: β-hydroxybutyrate.*
